# Determinants of long COVID among adults hospitalized for SARS-CoV-2 infection: A prospective cohort study

**DOI:** 10.3389/fimmu.2022.1038227

**Published:** 2022-12-19

**Authors:** Mattia Bellan, Daria Apostolo, Alice Albè, Martina Crevola, Nicolò Errica, Giacomo Ratano, Stelvio Tonello, Rosalba Minisini, Davide D’Onghia, Alessio Baricich, Filippo Patrucco, Patrizia Zeppegno, Carla Gramaglia, Piero Emilio Balbo, Giuseppe Cappellano, Sara Casella, Annalisa Chiocchetti, Elisa Clivati, Mara Giordano, Marcello Manfredi, Giuseppe Patti, David James Pinato, Chiara Puricelli, Davide Raineri, Roberta Rolla, Pier Paolo Sainaghi, Mario Pirisi, the No-More COVID study group, Mattia Bellan

**Affiliations:** ^1^ Università del Piemonte Orientale (UPO), Novara, Italy; ^2^ ”AOU Maggiore della Carità”, Novara, Italy; ^3^ Department of Surgery and Cancer, Imperial College London, Hammersmith Hospital, London, United Kingdom; ^4^ Dipartimento di Scienze della Salute, Novara, Italy

**Keywords:** long COVID-19, SARS-CoV-2 infection, cytokines, DLCO, depression

## Abstract

**Rationale:**

Factors associated with long-term sequelae emerging after the acute phase of COVID-19 (so called “long COVID”) are unclear. Here, we aimed to identify risk factors for the development of COVID-19 sequelae in a prospective cohort of subjects hospitalized for SARS-CoV-2 infection and followed up one year after discharge.

**Methods:**

A total of 324 subjects underwent a comprehensive and multidisciplinary evaluation one year after hospital discharge for COVID-19. A subgroup of 247/324 who consented to donate a blood sample were tested for a panel of circulating cytokines.

**Results:**

In 122 patients (37.8%) there was evidence of at least one persisting physical symptom. After correcting for comorbidities and COVID-19 severity, the risk of developing long COVID was lower in the 109 subjects admitted to the hospital in the third wave of the pandemic than in the 215 admitted during the first wave, (OR 0.69, 95%CI 0.51-0.93, p=0.01). Univariable analysis revealed female sex, diffusing capacity of the lungs for carbon monoxide (DLCO) value, body mass index, anxiety and depressive symptoms to be positively associated with COVID-19 sequelae at 1 year. Following logistic regression analysis, DLCO was the only independent predictor of residual symptoms (OR 0.98 CI 95% (0.96-0.99), p=0.01). In the subgroup of subjects with normal DLCO (> 80%), for whom residual lung damage was an unlikely explanation for long COVID, the presence of anxiety and depressive symptoms was significantly associated to persistent symptoms, together with increased levels of a set of pro-inflammatory cytokines: interferon-gamma, tumor necrosis factor-alpha, interleukin (IL)-2, IL-12, IL-1β, IL-17. In logistic regression analysis, depressive symptoms (p=0.02, OR 4.57 [1.21-17.21]) and IL-12 levels (p=0.03, OR 1.06 [1.00-1.11]) 1-year after hospital discharge were independently associated with persistence of symptoms.

**Conclusions:**

Long COVID appears mainly related to respiratory sequelae, prevalently observed during the first pandemic wave. Among patients with little or no residual lung damage, a cytokine pattern consistent with systemic inflammation is in place.

## 1 Introduction

The Severe Acute Respiratory Syndrome Coronavirus-2 (SARS-CoV-2) pandemic continues to have an impact on global health, as a result of acute infection and long-term sequelae. Unfortunately, a significant proportion of Coronavirus Disease (COVID-19) survivors experiences the persistence of symptoms many months after the acute phase of disease (1, 2).

Up to 58% of recovered patients show at least one symptom up to two years after the acute phase of the disease (3). The constellation of symptomatic and functional sequelae arising post-COVID-19 has been dubbed “long COVID”, thereby categorizing those patients who have symptoms that persist or develop after the acute phase of SARS-CoV-2 infection and are not explained by alternative diagnoses (4). Long COVID is a multi-systemic condition which encompasses pulmonary, immunological, cardiovascular, neuropsychiatric, gastroenterological, hepatic, renal, endocrine and dermatological sequelae (5–7).

Whereas the pathogenetic mechanisms that determine the acute phase of disease have been quite well elucidated (8), those underlying the long COVID syndrome remain poorly understood (9, 10). Indeed, a dysregulated inflammatory response with a huge release of pro-inflammatory cytokines, including IL-1β, IL-7, IL-8, IL-9, IFN-γ, TNF, has been observed during the acute phase of the disease (11–13). This condition may result in the recruitment of different immune cells at the site of infection leading to inflammatory damage of the alveolar-capillary membrane and vascular barrier damage inducing respiratory failure and the development and progression of acute respiratory distress syndrome (ARDS) (14, 15). Eventually, the uncontrolled immune response may result in extra-pulmonary manifestations with a possible evolution into multi-organ failure (11).

A variety of mechanisms have been claimed as potentially relevant for long COVID, among which the persistence of end organ damage following acute COVID-19, the effect of mood perturbation and the maintenance of a chronic pro-inflammatory state (5, 16, 17). The latter hypothesis belongs to the observation that long COVID shares clinical features with myalgic encephalomyelitis/chronic fatigue syndrome (ME/CFS) (18), a poorly understood clinical condition, which can be elicited by acute viral infections. Indeed, the evidence is growing to suggest the persistence of a pro-inflammatory state in long COVID, months after virus has been cleared from the organism and the convalescence phase has been completed (19, 20).

Further insight into the pathogenesis of long COVID is paramount to pave the way for better tailored treatments and to contain the demand on health systems still under heavy pressure for the pandemic (5). In the present paper, we aimed to evaluate the role of potentially relevant pathogenetic mechanisms in the development of long COVID: more specifically, we evaluated the potential role of residual organ damage, persistent inflammation and psychological impact. This is a novel and multidisciplinary approach allowing us to better verify the relative contribution of these pathogenetic mechanisms to the development of long term sequelae.

## 2 Materials and methods

This prospective cohort study was approved by the local ethics committee (Comitato Etico Interaziendale Novara, IRB code CE 117/20) and conducted in strict accordance with the principles of the Declaration of Helsinki. All participants signed a written informed consent.

### 2.1 Study population

We contacted patients discharged between March 1^st^, 2020 and May 28^th^, 2021, from the Maggiore della Carità University Hospital of Novara, Piedmont, with a confirmed diagnosis of COVID-19. We included all patients aged 18 years or older who agreed to participate to the study. Patients were divided into two subsets: the first one which included subjects discharged between March 1^st^ and June 29^th^, 2020, corresponding to the first wave of the pandemic in Italy; the second one, including patients admitted during the third Italian wave (15^th^ March, 28^th^ May 2021).

The patients were contacted by telephone and asked to attend a dedicated clinic for a follow-up visit 12 months after discharge; of them, 324 agreed to participate in the study. Follow-up visits took place from March 15^th^, 2021 to June 28^th^, 2022.

### 2.2 Study procedures

Patients were evaluated with a multidisciplinary approach, which included:

1. Clinical evaluation
2. Lung function assessment
3. Mental health assessment

#### 2.2.1 Clinical evaluation

A survey was carried out aimed at recording: patients’ demographic characteristics, symptoms during the acute phase of the disease, severity of the acute phase of the disease classified using an eight-category scale, as previously described (21), home medications and comorbidities which were cumulatively scored using the cumulative illness rating scale (CIRS) (22), patient symptoms at follow-up, vital signs and cardio-pulmonary physical examination.

#### 2.2.2 Lung function assessment

All patients underwent standard pulmonary function tests (PFT) with a Quark PFT with X9 pneumotach (COSMED srl, Rome, Italy) to evaluate forced expiratory volume in 1 second (FEV1), vital capacity, forced vital capacity (FVC), diffusing capacity of the lungs for carbon monoxide (DLCO), DLCO constant, and total lung capacity. DLCO and total lung capacity were determined by the single-breath co technique.

The spirometer was calibrated on the day the test was performed and the barometric pressure and temperature were simultaneously recorded. A trained technician coached the patient, while a pulmonologist was responsible for test validation and interpretation based on the 2005 the American Thoracic Society and European Respiratory Society statements. Appropriate safety measures were adopted (including use of a dedicated spirometer to avoid cross-infection, use of a mouthpiece with a different antimicrobial filter for each patient and use of complete PPE by the staff). At the end of each day, the room underwent disinfection.

#### 2.2.3 Mental health assessment

Patients were interviewed by an experienced psychiatrist, trained in the use of the Mini-International Neuropsychiatric Interview (MINI) (23), using structured and unstructured questions about current mental health status. Information about previous psychiatric history (being already treated by psychiatric services; a history of depression and/or anxiety), presence/absence of depressive and anxiety symptoms (independent of a full-criteria diagnosis of major depressive disorder, panic disorder, and generalized anxiety disorder) was gathered.

### 2.3 Laboratory analysis

To evaluate the potential role of cytokines, we performed a multipanel assessment of pro-inflammatory cytokines, using a Bio-PlexProTM Human Cytokine Assays (BioRad, Hercules, CA, US CAT.#M500KCAF0Y): interferon (IFN)-γ, interleukin (IL)-1β, IL-2, IL-6, IL-12, IL-17, tumor necrosis factor (TNF)-α were dosed. These studies were performed on 247/324 subjects who agreed to undergo peripheral venous blood sampling at the 12-months follow-up visit; for 73 patients, a blood sample, obtained during the acute phase of disease was also available and was therefore used to assess the potential predictive value of baseline cytokines levels on long COVID development.

Bio-PlexProTM Assays are sandwich immunoassays formatted on magnetic beads. Primitive antibodies directed against the desired biomarker are covalently coupled to the beads. Coupled beads react with the sample containing the biomarker of interest. After a series of washes to remove unbound protein, a biotinylated detection secondary antibody is added to create a sandwich complex. The final detection complex is formed with the addition of streptavidin-phycoerythrin (SA-PE) conjugate. Phycoerythrin serves as a fluorescent indicator, or reporter.

The samples were processed following the steps of the Bio-PlexProTM Assays manual issued by the supplier.

### 2.4 Statistical analysis

The data collected were recorded in a database and analyzed with MedCalc v.18.10.2 (MedCalc Software, Mariakerke, Belgium). For continuous variables, the measures of centrality and dispersion were medians and interquartile ranges [IQR] and the differences between groups were evaluated by the Mann-Whitney test. The Pearson χ^2^ or the Fisher exact test were used, as appropriate, to analyze the association between categorical variables. The existence of a correlation between continuous variables was verified calculating the corresponding Spearman correlation coefficient ρ. All variables found to have a statistical association with residual symptoms or DLCO impairment were entered into multivariate logistic regression models. The threshold for statistical significance was set at 0.05 (two-tailed).

## 3 Results

### 3.1 Impact of the pandemic wave on the development of long COVID

Out of the cohort of 324 subjects (196 males, 60.5%), 215 (66.3%) had been admitted to hospital during the first wave of the pandemic; data about their long-term sequelae have been published previously (18). Conversely, 109 were diagnosed during the third wave of the pandemic. In **Supplementary Table 1** we described the main features of the general population, during the acute phase of disease.

After 368 [364-391] days, 37.8% of patients (N. = 122) still complained at least one physical symptom (also see **Supplementary Table 2** for more details) and 151 (46.7%) patients reported the persistence of a reduction of their tolerance to physical activity.

Moreover, among the 310 patients who underwent a comprehensive psychiatric evaluation, anxiety symptoms were reported by 54 (17.4%) patients, while depressive symptoms were reported by 66 (21.3%) subjects.

Finally, 317 subjects underwent a pulmonary function test, according to which, the median FEV1 was 100 [89-112] of predicted, the median forced FVC was 80 [70-91] of predicted and the median DLCO was 98 [88-109] of predicted.

One of our research questions was to verify whether the risk of developing persistent symptoms might have changed over time. As shown in **Table 1**, the proportion of subjects complaining persistent symptoms was similar among those subjects admitted to the hospital during the first wave and those admitted during the third wave. However, in the third wave both the median severity of their condition and CIRS score were higher.

**Table 1:** General features of the study population classified according to the wave of hospital admission.

	Patients admitted during the first wave	Patients admitted during the third wave	p
**Age, years**	61 [50-70]	59 [52-67]	0.51
**Sex, M/F**	132 (61.4)/83 (38.6)	64 (58.7) /45 (41.3)	0.72
Oxygen supplementation			
** None** ** Nasal cannulae or Ventimask** ** Non-invasive ventilation** ** Mechanical ventilation**	63 (29.3) 91 (41.3) 43 (20.0) 18 (8.4)	0 (0.0) 24 (22.0) 85 (78.0) 0 (0.0)	<0.0001*
Class of severity			
** 3** ** 4** ** 5** ** 6** ** 7**	51 (23.7) 9 (4.2) 86 (40.0) 48 (22.3) 21 (9.8)	1 (0.9) 0 (0.0) 23 (21.1) 85 (78.0) 0 (0.0)	<0.0001*
**CIRS**	2 [1-3]	4 [2-6]	<0.0001*
**Residual symptoms, N/Y**	126 (58.9)/88 (41.1)	71 (65.1)/34 (31.2)	0.09
**Anxiety symptoms, N/Y**	163 (81.1)/38 (18.9)	93 (85.3)/16 (14.7)	0.43
**Depressive symptoms**	161 (80.1)/40 (19.9)	83 (76.1)/26 (23.9)	0.47
**FEV1, % of predicted**	100 [90-112]	101 [87-111]	0.46
**FVC, % of predicted**	98 [89-109]	98 [86-108]	0.64
**DLCO, % of predicted**	78 [68-90]	81 [72-93]	0.02

CIRS, cumulative illness rating scale; DLCO, diffusing capacity of the lungs for carbon monoxide; FEV1, forced expiratory volume in the first second; FVC, forced vital capacity. *p < 0.05

Therefore, to rule out the impact of the different time of hospital admission, we built a logistic regression model, which showed that being admitted to the hospital during the third wave was protective against the development of persistent symptoms (p=0.01, OR 0.69, 95%CI 0.51-0.93). Moreover, CIRS (p=0.22) and disease severity (p=0.13) during the acute phase were not associated to the development of persistent symptoms. However, in a multiple regression analysis model, the 12 months DLCO value was predicted by the wave of pandemic during which the patient was admitted to hospital (p<0.0001), along with disease severity during acute phase (p=0.02) and CIRS (p<0.0001).

### 3.2 Evaluation of pathogenetic mechanisms underlying the development of long COVID

To better evaluate the pathogenetic mechanisms underlying the development of long COVID, we analyzed a subgroup of 247 patients (151 males, 61.1%) with a median age of 60 [51-69] years, for whom a blood sample, collected one year after hospital discharge, was available. 155 (62.8%) were admitted during the first wave, while 92 (37.2%) were admitted during the third wave.

The median number of ongoing drugs for routine therapies at admission was 1 [0-4], while the median number of comorbidities was 2 [1-3], among them arterial hypertension (42.9%), obesity (22.2%) and diabetes mellitus (15.1%) were the most frequent ones, and the median CIRS was 2 [1-4].

The median duration of hospital stay during the acute phase was 11 [7-16] days. Disease severity during the acute phase was distributed as follows:

-Class 3, N. 37 (15.0%);
-Class 4, N. 5 (2.0%);
-Class 5, N. 83 (33.6%);
-Class 6, N. 107 (43.3%);
-Class 7, N. 15 (6.1%).

During hospital stay, 44 patients did not require oxygen supplementation (17.8%), 87 received oxygen through nasal cannula or Venturi mask (35.2%), 104 underwent non-invasive ventilation (42.1%) and, finally, 12 (4.9%) were mechanically ventilated. 18 (7.3%) were admitted to an intensive care unit, where they stayed for a median of 7.5 [5.0-20.0] days.

One year after discharge, 93 subjects (37.7%) still complained of at least one symptom (also see **Table 2** for more details). Moreover, 111 subjects (44.9%) reported a reduction of their tolerance to physical effort and 64 (25.9%) reported alopecia. The main symptoms and the results of pulmonary function tests are reported in **Table 2**.

**Table 2:** Main findings at the one year follow-up visit.

Cough	20 (8.1)
Fever	3 (1.2)
Dyspnoea	11 (4.5)
Diarrhoea	13 (5.3)
Arthralgia/Myalgia	41 (16.6)
Dysgeusia	15 (6.1)
Fatigue	38 (15.4)
Anosmia	19 (7.7)
DLCO	81 [70 – 91]
FEV1	102 [94,3 – 113,8]
FVC	98 [89 – 109]

DLCO, diffusing capacity of the lungs for carbon monoxide; FEV1, forced expiratory volume in the first second; FVC, forced vital capacity.

The association between clinical variables and persistence of symptoms is shown in **Table 3**, which identified DLCO, BMI, the persistence of anxiety and depressive symptoms and female sex as predictors at univariate analysis. We built a logistic regression analysis model including as independent variables all those associated to persistent symptoms at univariate analysis: DLCO emerged as the only independent predictor of residual symptoms (OR 0.98 CI95% (0.96-0.99), p=0.01), suggesting the relevance of residual organ impairment in the development of long COVID (**Supplementary Table 3**).

**Table 3:** Univariate analysis of clinical variables among patients with and without residual symptoms.

	No residual symptoms	Residual symptoms	p
Age, years	59 [51-68]	61 [50-70]	0.63
Gender, M/F	106 (70.2)/48 (50.0)	45 (29.8)/48 (50.0)	0.002*
Oxygen supplementation			
None Nasal cannulae or Ventimask Non-invasive ventilation Mechanical ventilation	25 (16.2) 60 (39.0) 61 (39.6) 8 (5.2)	19 (20.4) 27 (29.0) 43 (46.2) 4 (4.3)	0.41
Class of severity			
3 4 5 6 7	22 (14.3) 3 (1.9) 56 (36.4) 65 (42.2) 8 (5.2)	15 (16.1) 2 (2.2) 27 (29.0) 42 (45.2) 7 (7.5)	0.79
Oxygen supplementation			
None Nasal cannula or Venturi mask Non-invasive ventilation Mechanical ventilation	39 (19.4) 76 (37.8) 76 (37.8) 10 (5.0)	24 (19.7) 38 (31.1) 52 (42.6) 8 (6.6)	0.63
Drugs			
Hydroxychloroquine Steroids Antiviral agents Anti-IL6	106 (57.0) 87 (46.8) 76 (40.9) 5 (2.7)	75 (66.4) 46 (40.7) 52 (46.0) 3 (2.7)	0.11 0.34 0.40 1.00
CIRS	2 [1-4]	2 [2-3]	0.45
BMI, kg/m2	27.4 [24.7-31.2]	30.1 [26.2-32.3]	0.02*
Anxiety symptoms, N/Y	136 (89.5)/16 (10.5)	67 (77.0)/20 (23.0)	0.01*
Depressive symptoms, N/Y	128 (84.2)/24 (15.8)	59 (67.8)/28 (32.2)	0.005*
FEV1, %	102 [88-111]	100 [90-114]	0.98
FVC, %	98 [89-109]	97 [87-108]	0.42
DLCO, %	83 [72-91]	77 [68-84]	0.0007*

BMI, body mass index; CIRS, cumulative illness rating scale; DLCO, diffusing capacity of the lungs for carbon monoxide; FEV1, forced expiratory volume in the first second; FVC, forced vital capacity. *p < 0.05.

This hypothesis is supported by the demonstration that the prevalence of residual symptoms is higher among the 111 subjects with a reduction of DLCO below the threshold of 80% of predicted (N. = 53, 47.7%), than in those with a normal DLCO (N. = 40, 30.1%; p=0.005).

However, this finding also confirms that a consistent proportion of subjects with no significant respiratory function impairment still complains symptoms 1 year after hospital discharge. We therefore investigated other potential pathogenetic factors, firstly focusing our attention on the psychiatric sequelae. To do that, we investigated the association between anxiety/depression symptoms and long COVID among patients with and without a reduction of DLCO (**Table 4**). As shown in the table, the presence of anxiety or depression symptoms is associated with long COVID only among those subjects who did not show a significant reduction in their DLCO, suggesting that in this subset, mental health may play a key role in the development of long COVID.

**Table 4:** Proportion of patients complaining residual symptoms and mental health impairment categorized according to diffusing capacity of the lungs for carbon monoxide (DLCO) values.

DLCO ≥ 80%			
	**No residual symptoms**	**Residual symptoms**	**p**
**Anxiety symptoms, No/Yes**	84 (91.3)/8 (8.7)	27 (71.1)/11 (28.9)	0.003*
**Depressive symptoms, No/Yes**	83 (90.2)/9 (9.8)	23 (60.5)/15 (39.5)	0.0002*
DLCO < 80%			
	**No residual symptoms**	**Residual symptoms**	**p**
**Anxiety symptoms, No/Yes**	50 (87.7)/7 (12.3)	40 (81.6)/9 (18.4)	0.42
**Depressive symptoms, No/Yes**	44 (77.2)/13 (22.8)	36 (73.5)/13 (26.5)	0.82

*p < 0.05.

We finally aimed to evaluate the potential role of a persistent subclinical inflammation assessed with the dosage of a set of pro-inflammatory cytokines: IL-2, IL-12, IL-1β, IFN-γ, TNF-α and IL-17. In **Supplementary Table 4** we report the plasma levels of these cytokines in the study population; moreover, we report the concentrations observed in a subgroup of 73 subjects for whom a baseline blood sample, obtained during the acute phase of disease was available. We then evaluated whether the persistence of symptoms was associated to cytokine levels at baseline or at follow-up visit (**Table 5**). As reported in the table, none of the assessed cytokines, measured at baseline, predicted the development of persistent symptom. Conversely, patients suffering of long COVID, showed increased values of IL-12, IL-17, IL-1β, IFN-γ and TNF-α, one year after hospital discharge.

**Table 5:** Levels of pro-inflammatory cytokines in patients with and without residual symptoms.

Cytokine concentration at baseline (N. = 73)	Residual symptoms	No residual symptoms	p
**IL-12**	3.47 [0.12-5.73]	2.62 [0.00-4.68]	0.47
**IL-1ß**	0.59 [0.07-2.57]	0.41 [0.00-1.26]	0.38
**IL-17**	5.43 [2.63-8.72]	3.93 [2.54-5.43]	0.07
**IL-6**	8.03 [4.92-25.28]	9.12 [5.35-19-51]	0.95
**IL-2**	2.16 [0.00-6.57]	2.16 [0.00-3.84]	0.49
**IFN-γ**	6.09 [5.20-12.03]	7.64 [5.20-8.68]	0.87
**TNF-α**	21.36 [15.61-28.44]	20.15 [16.31-25.97]	0.86
**Cytokine concentration at follow-up visit (N. = 247)**	**Residual symptoms**	**No residual symptoms**	**p**
**IL-12**	12,90 [1.75-26.15]	4,40 [0.30-14.19]	0.0003*
**IL-1ß**	0,61 [0.00-1.69]	0,20 [0.00-0.80]	0.005*
**IL-17**	10,68 [5.25-25.04]	6,55 [3.24-13.81]	0.0008*
**IL-6**	0,00 [0.00-1.52]	0,00 [0.00-1.45]	0.65
**IL-2**	1,11 [0.00-3.43]	0,00 [0.00-1.19]	0.005*
**IFN-γ**	5,27 [2.03-15.14]	3,33 [1.69-8.71]	0.006*
**TNF-α**	53,70 [24.28-76.18]	43,57 [11.86-63.98]	0.01*

IFN, interferon; IL, interleukin; TNF, tumor necrosis factor. *p < 0.05.

In a logistic regression analysis, including clinical variables associated to the persistence of long-term sequelae, DLCO (OR 0.98, 95%CI 0.96-0.99; p=0.04) and IL-12 (OR 1.04, 95%CI 1.00-1.08; p=0.04) were the only variables independently associated with the persistence of symptoms at one year from hospital discharge in this subgroup (see **Supplementary Table 5**).

Interestingly, when we evaluated only those patients with a DLCO >80% and with no residual organ damage, we confirmed the independent association between residual symptoms and IL-12 plasma concentration (OR 1.06, 95%CI 1.00-1.11; p=0.03), which was associated with long COVID along with the persistence of depressive symptoms (OR 4.57, 95%CI 1.21-17.21; p=0.02) see **Supplementary Table 6**). Taking together these findings, IL-12 seems to be independently associated to the presence of persistent symptoms in patients with no significant organ involvement. Accordingly, IL-12 levels bore no correlation with DLCO (ρ=-0.079, p=0.22).

We finally evaluated whether any of the assayed cytokines may play a role in the development of alopecia; in **Table 6** we report the clinical features and the median values of cytokines concentration at baseline and at follow-up. As reported in the table, female sex, anxiety and depressive symptoms were associated with development of alopecia. The baseline levels of pro-inflammatory cytokines did not predict alopecia; in contrast, the concentration of IL-12, IL-2, IL-17, IFN-γ and TNF-α at 1 year was significantly increased in patients complaining alopecia. At logistic regression analysis, female sex and IL-17 plasma concentrations, were independently associated with development of alopecia (**Supplementary Table 7**).

**Table 6:** Comparison of clinical features and cytokine levels among patients with or without alopecia.

	Alopecia N = 64	Alopecia N = 180	p
**Age, years**	61 [50-69]	59 [51-68]	0.36
**Sex, M/F**	18 (11.9)/46 (47.9)	133 (88.1)/50 (52.1)	<0.0001*
**BMI, kg/m^2^**	28.6 [24.8-32.1]	28.1 [25.5-31.5]	0.92
**DLCO, %**	76 [68-85]	82 [70-92]	0.04*
**CIRS**	2 [1-3]	2 [1-4]	0.12
**Class of severity, 3/4/5/6/7**	11 (17.2)/1 (1.6)/23 (35.9)/22 (34.4)/7 (10.9)	26 (14.2)/4 (2.2)/60 (32.8)/85 (46.4)/8 (4.4)	0.24
**Anxiety symptoms, No/Yes**	43 (21.2)/16 (44.4)	160 (88.9)/20 (55.6)	0.005*
**Depressive symptoms, No/Yes**	39 (20.9)/20 (38.5)	148 (79.1)/32 (61.5)	0.02*
**IL-12 baseline**	3.47 [0.00-4.50]	2.62 [0.12-4.70]	0.66
**IL-1β baseline**	1.09 [0.15-2.33]	0.35 [0.00-1.43]	0.38
**IL-17 baseline**	3.33 [2.03-5.05]	4.37 [2.91-6.45]	0.07
**IL-6 baseline**	5.35[2.13-22.81]	9.12 [5.39-20.62]	0.95
**IL-2 baseline**	2.46 [0.41-6.17]	2.16 [0.00-4.06]	0.49
**IFN-γ baseline**	5.60 [5.20-7.68]	7.37 [5.20-11.38]	0.87
**TNF-α baseline**	25.97 [20.45-27.79]	20.02 [15.42-27.88]	0.33
**IL-12 1-y follow-up**	12.44 [1.84-25.76]	5.18 [0.48-16.10]	0.007*
**IL-1β 1-y follow-up**	0.53 [0.00-1.61]	0.20 [0.00-0.84]	0.005*
**IL-17 1-y follow-up**	13.90 [7.51-25.72]	6.44 [3.24-13.81]	0.0008*
**IL-6 1-y follow-up**	0,00 [0.00-1.12]	0,00 [0.00-1.70]	0.65
**IL-2 1-y follow-up**	1,13 [0.00-3.85]	0,00 [0.00-1.52]	0.005*
**IFN-γ 1-y follow-up**	6.26 [2.76-16.04]	3.29 [1.69-8.71]	0.002*
**TNF-α 1-y follow-up**	56.95 [46.57-73.66]	41.76[12.68-64.03]	0.0007*

BMI, body mass index; CIRS, cumulative illness rating scale; DLCO, diffusing capacity of the lungs for carbon monoxide; IFN, interferon; IL, interleukin; TNF, tumor necrosis factor. *p < 0.05.

## 4 Discussion

The present study shows that the risk of long COVID was significantly greater in the first wave of pandemic, when compared to the third one. Moreover, we confirm that respiratory function impairment, assessed by DLCO measurement, is the main factor associated with the persistence of symptoms. This suggests that residual organ damage plays a key role in the development of residual symptoms; however long COVID may affect also patients with normal lung function. Among those subjects with preserved respiratory function, our results suggest that the development of depressive symptoms and the persistence of a chronic inflammatory state may represent major determinants of long COVID. These novel findings deserve a deeper discussion.

In the present study we firstly considered a large cohort of 324 patients followed-up, with a multidisciplinary approach, one year after their discharge from an Italian hospital. Part of the present cohort had already been described in a previous paper (24). Consistently with our previous findings, around 38% of patients still complained symptoms one year after the acute phase of disease. This is in line with previous papers (25, 26), which reported similar rates of long COVID at the same timepoint. Other authors reported even higher rates of residual symptoms, although differences in clinical assessment and in the definition of long COVID may easily subside the discrepancies with our findings (3, 27).

Our first aim was to evaluate whether being admitted at different waves of the pandemic was associated to a different risk of residual symptoms. Indeed, it is well known that the case fatality rate of the acute phase of disease is decreasing over time (28, 29). However, it is still unclear whether this outcome improvement might also be associated to a reduced risk of developing long COVID. Despite finding a similar proportion of subjects with residual symptoms, the patients admitted during the third wave were more comorbid and showed a higher class of disease severity during the acute phase. Indeed, during the first wave of the pandemic in Italy, when the virus was largely unknown and the number of cases relatively low, there was a local tendency of admitting a larger proportion of patients who tested positive, even when there was no evidence of respiratory failure. Conversely, during the third wave, the huge number of patients testing positive and requiring oxygen supplementation prevented the admission of patients with mild disease.

When we assessed the role of the wave of pandemic on long COVID development, we demonstrated that, being admitted during the third wave was protective against long COVID; this observation, to the best of our knowledge, is novel and probably reflects the improvement of preventive (vaccines) and therapeutic strategies. Although a direct effect related to the changes of the virus, with the development of subsequent variants, can only be hypothesized, it should be acknowledged that patients in the third wave were infected when delta variant was the most represented one in Italy. It is well known that this specific variant was burdened by a severity and mortality even greater than alpha variant (30). In this context, a major limitation of our study is that we did not identify the variants affecting our patients. However, based on our findings, it is reasonable to postulate that, beside a reduction of the severity of acute infection, the prevalence of long-term sequelae may reduce over time.

We then moved our attention towards the identification of clinical factors associated with the development of long COVID. In line with previous reports, we found that a decreasing DLCO (31), female gender (32), a higher BMI (33) and the presence of anxiety and depression symptoms (34) were all associated to the persistence of symptoms at univariable analysis; when included in a logistic regression model, the strongest and unique independent predictor of residual symptoms was DLCO, suggesting that the residual organ impairment after acute phase might be the major driver of long COVID. Indeed, residual symptoms are, according to our data, more prevalent among patients with a DLCO<80%. Obviously, our data rely only on post-COVID PFTs; pre-COVID DLCO values were not available and, therefore, we are not able to assess whether this functional impairment should completely be attributed to SARS-CoV-2 infection or, rather, whether this might follow a pre-existing lung condition. Whichever the cause of DLCO impairment, the decrease in lung function remains the most relevant factor associated with long COVID symptoms.

Clearly, DLCO impairment cannot entirely justify the whole spectrum of long COVID; it is a common experience to identify patients with residual symptoms and a fully normal lung function. However, impaired DLCO may act as a surrogate for severe COVID-19, a condition known to be associated with a higher risk of sequelae compared to asymptomatic infection. In this context, other pathogenetic mechanisms should be involved.

First, we demonstrated that mental health impairment is very relevant in this subset of patients. Beside a strong association between anxiety/depressive and organic symptoms in patients with a DLCO > 80%, in those with a normal DLCO, this association was completely lost. This observation suggests that the mental health impact may be a cause, rather than a consequence of long COVID, at least for those patients without long term organ function impairment. It is well known that the prevalence of COVID-19 survivors showing anxiety/depressive symptoms is high (35, 36). The pandemic impacted severely on the mental health of the general population: the effects on persons who required hospital admission, especially if they experienced traumatic events such as mechanical ventilation or familiar mourns, are conceivably more severe. Our findings suggest that a proper psychological support might be helpful in preventing and/or attenuating long COVID.

We finally explored the hypothesis that a persistent pro-inflammatory state may contribute to long COVID pathogenesis. This idea stems from the observation that long COVID shares common features with other post-acute syndromes following viral infection, which have been linked to persistent chronic inflammation (37). The present paper supports this theory, since patients complaining persistent symptoms were characterized by increased concentration of a plethora of pro-inflammatory cytokines. Interestingly, the only one which was not associated was IL-6, which is known to have a key role during acute infection being a potential target of treatment (38). Conversely, we found that increased levels of IL-1β, IL-2, IL-12, IL-17, IFN-γ and TNF-α are associated with persistent symptoms of disease. Our findings integrate and expand recent data: in a paper by Queiroz et al. (39), IL-17 and IL-2 were reported to be increased in patients with post-acute sequelae. Similarly, Schultheiß recently reported the association of IL-1, IL-6 and TNFα (40). All these contributions point towards the hypothesis that counteracting this derangement of innate immunity might be helpful in the pharmaceutical management of long COVID. A novel finding emerging from our cohort is the central role of IL-12. IL-12 is a key cytokine in innate immunity, which enhances NK cytotoxicity and IFN-γ production (41). The association between IL-12 levels and long COVID has never been reported to date; however, in the past, increased levels of IL-12 have already been reported in patients with a condition bearing similarities with long COVID, i.e., CFS.

We finally focused our attention on a specific long-lasting condition, alopecia, which is unexpectedly reported in a high proportion of COVID-19 survivors (42). Previous data on alopecia areata suggested that cytokines may contribute to hair loss (43). Indeed, drugs counteracting cytokines activity, such as baricitinib are sometime used for alopecia areata treatment (44). However, to the best of our knowledge, the association between pro-inflammatory cytokines and alopecia in long COVID has never been shown before. According to our data, Th1 and Th17 cytokines are increased in patients complaining hair loss; among them, IL-17 seems to be particularly relevant as already reported by previous papers on alopecia areata (45).

Finally, it is relevant to underline that we also tried to evaluate the predictive role of baseline cytokines on the persistence of symptoms; we failed to disclose any association, although the small sample size of patients with the availability of a baseline blood sample might have underpowered our findings.

## 5 Limitations

We are aware that this study has some limitations. The small number of samples collected during the acute phase of disease prevent us to reliably evaluate the impact of acute inflammatory response on chronic disease. In addition, hospitalized patients represent a small proportion of COVID-19 cases and for this reason, focusing only on discharged hospitalized patients could provide a limited perspective on the experience of long COVID in the broader population. Moreover, we did not have specific information on the variants affecting our study population.

## 6 Conclusions

In conclusion, our work shades new light on the mechanisms underlying the development of long COVID, suggesting that the persistence of respiratory function impairment is crucial, but even in subjects with a completely normal lung function, symptoms may persist as a result of mental health derangement and/or persistent chronic inflammation, involving Th1 and Th17 cytokines. These findings, if confirmed in larger studies, might provide a rationale for trials with drugs targeting pro-inflammatory cytokines in patients with severe sequelae of SARS-CoV-2 infection.

## No-More COVID study group

Mattia Bellan, Università del Piemonte Orientale (UPO), Novara, Italy, “AOU Maggiore della Carità”, Novara, Italy; Daria Apostolo, Università del Piemonte Orientale (UPO), Novara, Italy; Alice Albè, Università del Piemonte Orientale (UPO), Novara, Italy, “AOU Maggiore della Carità”, Novara, Italy; Martina Crevola, Università del Piemonte Orientale (UPO), Novara, Italy, “AOU Maggiore della Carità”, Novara, Italy; Nicolò Errica, Università del Piemonte Orientale (UPO), Novara, Italy, “AOU Maggiore della Carità”, Novara, Italy; Giacomo Ratano, Università del Piemonte Orientale (UPO), Novara, Italy, “AOU Maggiore della Carità”, Novara, Italy; Acquaviva Antonio, Università del Piemonte Orientale (UPO), Novara, Italy, “AOU Maggiore della Carità”, Novara, Italy; Luigi Mario Castello, Università del Piemonte Orientale (UPO), Novara, Italy; Donato Colangelo, Dipartimento di Scienze della Salute, Novara, Italy; Stelvio Tonello, Università del Piemonte Orientale (UPO), Novara, Italy; Rosalba Minisini, Università del Piemonte Orientale (UPO), Novara, Italy; Davide D’Onghia, Università del Piemonte Orientale (UPO), Novara, Italy; Alessio Baricich, Università del Piemonte Orientale (UPO), Novara, Italy, “AOU Maggiore della Carità”, Novara, Italy; Filippo Patrucco, Università del Piemonte Orientale (UPO), Novara, Italy, “AOU Maggiore della Carità”, Novara, Italy; Patrizia Zeppegno, Università del Piemonte Orientale (UPO), Novara, Italy, “AOU Maggiore della Carità”, Novara, Italy; Carla Gramaglia, Università del Piemonte Orientale (UPO), Novara, Italy, “AOU Maggiore della Carità”, Novara, Italy; Gian Carlo Avanzi, Università del Piemonte Orientale (UPO), Novara, Italy, “AOU Maggiore della Carità”, Novara, Italy; Piero Emilio Balbo, “AOU Maggiore della Carità”, Novara, Italy; Giulia Baldon, Università del Piemonte Orientale (UPO), Novara, Italy, “AOU Maggiore della Carità”, Novara, Italy; Michela Barini, Università del Piemonte Orientale (UPO), Novara, Italy; Marco Battaglia, Università del Piemonte Orientale (UPO), Novara, Italy, “AOU Maggiore della Carità”, Novara, Italy; Simone Bor, Università del Piemonte Orientale (UPO), Novara, Italy, “AOU Maggiore della Carità”, Novara, Italy; Vincenzo Cantaluppi, Università del Piemonte Orientale (UPO), Novara, Italy, “AOU Maggiore della Carità”, Novara, Italy; Giuseppe Cappellano, “AOU Maggiore della Carità”, Novara, Italy; Alessandro Carriero, Università del Piemonte Orientale (UPO), Novara, Italy, “AOU Maggiore della Carità”, Novara, Italy; Sara Casella, Università del Piemonte Orientale (UPO), Novara, Italy; Annalisa Chiocchetti, Università del Piemonte Orientale (UPO), Novara, Italy; Elisa Clivati, “AOU Maggiore della Carità”, Novara, Italy; Daria Cuneo, Università del Piemonte Orientale (UPO), Novara, Italy, “AOU Maggiore della Carità”, Novara, Italy; Eleonora Gambaro, Università del Piemonte Orientale (UPO), Novara, Italy, “AOU Maggiore della Carità”, Novara, Italy; Mara Giordano, Università del Piemonte Orientale (UPO), Novara, Italy, “AOU Maggiore della Carità”, Novara, Italy; Luisa Isabella, Università del Piemonte Orientale (UPO), Novara, Italy, “AOU Maggiore della Carità”, Novara, Italy; Alberto Loro, Università del Piemonte Orientale (UPO), Novara, Italy, “AOU Maggiore della Carità”, Novara, Italy; Marcello Manfredi, Università del Piemonte Orientale (UPO), Novara, Italy; Debora Marangon, “AOU Maggiore della Carità”, Novara, Italy; Emanuele Mones, Università del Piemonte Orientale (UPO), Novara, Italy, “AOU Maggiore della Carità”, Novara, Italy; Elena Paracchini, “AOU Maggiore della Carità”, Novara, Italy; Giuseppe Patti, Università del Piemonte Orientale (UPO), Novara, Italy, “AOU Maggiore della Carità”, Novara, Italy; David James Pinato, Università del Piemonte Orientale (UPO), Novara, Italy, Department of Surgery and Cancer, Imperial College London, Hammersmith Hospital, London, United Kingdom; Chiara Puricelli, Università del Piemonte Orientale (UPO), Novara, Italy, “AOU Maggiore della Carità”, Novara, Italy; Davide Raineri, Università del Piemonte Orientale (UPO), Novara, Italy; Roberta Rolla, Università del Piemonte Orientale (UPO), Novara, Italy; Pier Paolo Sainaghi, Università del Piemonte Orientale (UPO), Novara, Italy, “AOU Maggiore della Carità”, Novara, Italy; Stefano Tricca, Università del Piemonte Orientale (UPO), Novara, Italy; Mario Pirisi, Università del Piemonte Orientale (UPO), Novara, Italy, “AOU Maggiore della Carità”, Novara, Italy.

## Data availability statement

The raw data supporting the conclusions of this article will be made available by the authors, without undue reservation.

## Ethics statement

The studies involving human participants were reviewed and approved by Comitato Etico Interaziendale Novara, IRB code CE 117/20. The patients/participants provided their written informed consent to participate in this study.

## Author contributions

MB had full access to all of the data in the study and takes responsibility for the integrity of the data and the accuracy of the data analysis. Study concept and design: MB, AB, FP, PZ, CG, GP, and MP. Acquisition of data: AAl, MC, NE, GR, AAn, AB, FP, PZ, CG, PB, SC, EC, GP, CP, and PS. Analysis and interpretation of data: MB, DA, ST, RM, DD, GC, AC, MG, MM, DR, and RR. Drafting of the manuscript: MB and DP. Critical revision of the manuscript for important intellectual content: All the authors. Statistical analysis: MB and MP. Obtained funding: MB and AC. Administrative, technical, or material support: ST and RM. Study supervision: MP. All authors contributed to the article and approved the submitted version.
