# Supplementary material for: Determinants of long COVID among adults hospitalized for SARS-CoV-2 infection: A prospective cohort study
Source: Front Immunol. 2022 Dec 19;13:1038227. doi: 10.3389/fimmu.2022.1038227 (PMC9807078; doi:10.3389/fimmu.2022.1038227)
Supplement: Supplementary file 1 [file DataSheet_1.docx]

Supplementary Material

# Supplementary Figures and Tables Supplementary material

## Supplementary table 1. General features of the whole study population. Abbreviations: CIRS, cumulative illness rating scale.

| Age, years | 60 [50-69] |
| --- | --- |
| Oxygen supplementation, none/nasal cannula or Venturi mask / Non invasive ventilation / mechanical ventilation | 63 (19.4) / 115 (35.5) / 128 (39.5) / 18 (5.6) |
| Class of severity, 3 / 4 / 5 / 6 / 7 | 52 (16.0) / 9 (2.8) / 109 (33.6) / 133 (41.0) / 21 (6.5) |
| CIRS | 2 [1-3] |
| Duration of hospital in stay | 10 [7-16] |

## Supplementary table 2. Frequency of the symptoms complained one year after hospital discharge

| Cough | 28 (8.7) |
| --- | --- |
| Fever | 4 (1.2) |
| Dyspnoea | 18 (5.6) |
| Diarrhoea | 19 (5.9) |
| Arthralgia/Myalgia | 58 (18.0) |
| Dysgeusia | 17 (5.3) |
| Fatigue | 52 (16.1) |
| Anosmia | 24 (7.4) |

## Supplementary table 3. Logistic regression analysis including variables associated to the persistence of long-term symptoms. Abbreviations: BMI, body mass index; DLCO, diffusing capacity of the lungs for carbon monoxide.

| Variable | Coefficient | OR (95%CI) | p |
| --- | --- | --- | --- |
| Sex | 0.51 | 1.67 (0.91-3.06) | 0.10 |
| DLCO | -0.02 | 0.98 (0.96-0.99) | 0.01* |
| BMI | 0.05 | 1.04 (0.99-1.10) | 0.08 |
| Anxiety symptoms | 0.64 | 1.90 (0.79-4.57) | 0.15 |
| Depressive symptoms | 0.48 | 1.62 (0.74-3.53) | 0.22 |

## Supplementary table 4. Plasma concentration of pro-inflammatory cytokines at baseline and at 1-year follow-up. Abbreviations: IFN, interferon; IL, interleukin; TNF, tumor necrosis factor.

|  | **Baseline**  **N.73** | **1-year follow-up**  **N. 274** |
| --- | --- | --- |
| IL-1β | 0.47 [0.00-1.49] | 0.26 [0.00-1.10] |
| IL-2 | 2.16 [0.00-4.13] | 0.19 [0.00-2.29] |
| IL-6 | 8.62 [5.20-21.07] | 0.00 [0.00-1.45] |
| IL-12 | 2.62 [0.09-4.70] | 7.57 [0.82-19.31] |
| IL-17 | 4.19 [2.54-6.37] | 8.04 [3.45-17.24] |
| IFN-γ | 7.37 [5.20-10.89] | 3.79 [1.69-12.42] |
| TNF-α | 20.15 [15.98-27.88] | 47.76 [15.91-66.50] |

## Supplementary table 5. Logistic regression analysis of variables associated with the persistence of symptoms. Abbreviations: BMI, body mass index; DLCO, diffusing capacity of the lungs for carbon monoxide, IFN, interferon; IL, interleukin; TNF, tumor necrosis factor.

| Variable | Coefficient | OR (95%CI) | p |
| --- | --- | --- | --- |
| Sex | 0.44 | 1.56 (0.83-2.93) | 0.17 |
| DLCO | -0.02 | 0.98 (0.96-0.99) | 0.04* |
| BMI | 0.05 | 1.05 (0.99-1.10) | 0.08 |
| Anxiety symptoms | 0.58 | 1.79 (0.71-4.50) | 0.21 |
| Depressive symptoms | 0.43 | 1.54 (0.68-3.46) | 0.33 |
| IL-12 | 0.04 | 1.04 (1.00-1.08) | 0.04* |
| IL-17 | 0.04 | 1.04 (0.99-1.09) | 0.15 |
| IL-2 | -0.06 | 0.94 (0.84-1.04) | 0.25 |
| IL-1β | 0.58 | 0.98 (0.82-1.18) | 0.86 |
| IFN-γ | 0.01 | 1.01 (0.97-1.05) | 0.52 |
| TNF-α | -0.01 | 0.99 (0.82-1.18) | 0.25 |

# Supplementary table 6 Logistic regression analysis of variables associated with the persistence of symptoms among patients with normal DLCO. Abbreviations: BMI, body mass index; DLCO, diffusing capacity of the lungs for carbon monoxide, IFN, interferon; IL, interleukin; TNF, tumor necrosis factor.

| Variable | Coefficient | OR (95%CI) | p |
| --- | --- | --- | --- |
| Sex | 0.22 | 1.25 (0.40-3.82) | 0.70 |
| DLCO | -0.04 | 0.96 (0.91-1.00) | 0.10 |
| BMI | 0.05 | 1.05 (0.91-1.15) | 0.22 |
| Anxiety symptoms | 0.85 | 2.33 (0.59-9.28) | 0.23 |
| Depressive symptoms | 1.51 | 4.57 (1.21-17.21) | 0.02* |
| IL-12 | 0.06 | 1.06 (1.00-1.12) | 0.03* |
| IL-17 | 0.07 | 1.08 (0.96-1.21) | 0.21 |
| IL-2 | -0.01 | 0.99 (0.88-1.10) | 0.80 |
| IL-1β | -0.18 | 0.83 (0.58-1.19) | 0.32 |
| IFN-γ | -0.01 | 0.99 (0.87-1.12) | 0.86 |
| TNF-α | -0.01 | 0.99 (0.97-1.01) | 0.40 |

## Supplementary table 7 Logistic regression analysis of factors associated to alopecia. Abbreviations: IFN, interferon; IL, interleukin; TNF, tumor necrosis factor.

| Variable | Coefficient | OR (95%CI) | p |
| --- | --- | --- | --- |
| Gender | 1.84 | 6.27 (3.00-13.08) | <0.0001* |
| Anxiety symptoms | 0.66 | 1.93 (0.74-5.02) | 0.18 |
| Depressive symptoms | -0.23 | 0.79 (0.32-1.92) | 0.61 |
| IL-12 | 0.02 | 1.02 (0.98-1.05) | 0.27 |
| IL-17 | 0.06 | 1.06 (1.01-1.11) | 0.01* |
| IL-2 | -0.001 | 0.99 (0.92-1.07) | 0.80 |
| IL-1β | -0.18 | 0.84 (0.61-1.14) | 0.26 |
| IFN-γ | 0.01 | 1.01 (0.98-1.05) | 0.43 |
| TNF-α | -0.002 | 0.99 (0.98-1.01) | 0.79 |

## Supplementary Figure 1. Heat map of 1-y follow-up cytokines levels in patients with and without residual symptoms. Pro-inflammatory cytokines concentration is normalized using the corresponding level in patients with no residual symptoms.

#
